# Primary Philadelphia chromosome positive acute myeloid leukemia: A case report

**DOI:** 10.1097/MD.0000000000012949

**Published:** 2018-11-02

**Authors:** Xiaoyan Shao, Dangui Chen, Peipei Xu, Miaoxin Peng, Chaoyang Guan, Pinhao Xie, Cuiying Yuan, Bing Chen

**Affiliations:** a Department of Hematology, Nanjing Drum Tower Hospital, The Affiliated Hospital of Nanjing University Medical School; b Department of Hematology, Nanjing Drum Tower Hospital, Clinical College of Nanjing Medical University, Nanjing, People's Republic of China.

**Keywords:** acute myeloid leukemia, dasatinib, Philadelphia chromosome

## Abstract

**Rationale:**

Philadelphia chromosome positive acute myeloid leukemia (Ph+ AML) is a rare subtype of AML that is now included as a provisional entity in the 2016 revised WHO classification of myeloid malignancies. However, a clear distinction between de novo Ph+ AML and chronic myeloid leukemia blast crisis is challenging. It is still a matter of debate whether Ph+ AML patients should be treated with chemotherapy or tyrosine kinase inhibitors as first-line therapy.

**Patient concerns:**

We reported here a case of a 46-year-old man who was diagnosed as Ph+ AML. This diagnosis was confirmed by bone marrow pathology and karyotype analysis of 46, XY, *t* (9; 22). Further examination, molecular genetic analysis showed BCR/ABL1 (p190) without ABL1 kinase domain mutations, and direct evidence demonstrated in AML by flow cytometry.

**Diagnosis:**

The diagnosis of Ph+ AML was made on May 2016 according to morphology, immunology, cytogenetic, and molecular criteria, and multiple organ failure was also diagnosed.

**Interventions:**

The patient was treated with dasatinib as the only medication after experiencing multiple organ failure. Then, he received 2 cycles of chemotherapy with IA (idarubicin 8 mg/m^2^, day 1–3; cytarabine 100 mg/m^2^, day 1–7) in August, 2016.

**Outcomes:**

The patient finally achieved a complete molecular remission.

**Lessons:**

This case study suggests that dasatinib can be a safe and effective treatment for Ph+ AML patients with poor physical condition.

## 1 Introduction

Philadelphia chromosome is the result of a reciprocal translocation involving the long arms of chromosomes 9 and 22, referred to as *t* (9; 22) (q34; q11). A hallmark of chronic myeloid leukemia (CML), and it is also common in patients with acute lymphoblastic leukemia and mixed phenotype acute leukemia.^[1,2]^ The incidence of Philadelphia chromosome in de novo acute myeloid leukemia (AML) ranges from 0.5% to 3%.^[3–7]^ Although it became a provisional entity of myeloid neoplasms and acute leukemia in the 2016 revised WHO classification,^[8]^ the diagnostic criteria and standard treatment for Philadelphia chromosome positive AML (Ph+ AML) remained unclear due to limited literature. Recently, a review described the current report of Ph+ AML clinical features and treatments compared to chronic myeloid leukemia primary in blast crisis (CML-BC).^[9]^ It appeared that Ph+ AML presented basophils <2% in white blood cell (WBC), p190 positive and <100% BCR/ABL1 metaphases and there was no standardized treatment. This case reported clinical features of Ph+ AML, and showed that dasatinib followed by chemotherapy treatment could help patients achieve complete molecular remission. This regimen was suggested to be a safe and effective way for Ph+ AML patients who were in and unable to receive chemotherapy as first-line therapy because of a poor physical condition.

## 2 Case presentation

**Figure 1:**
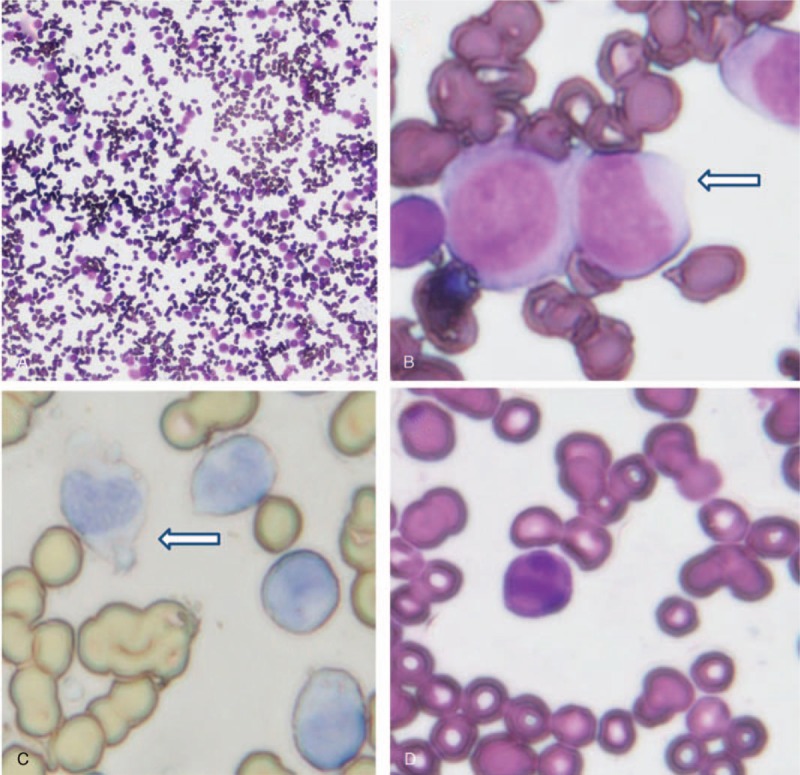
The patient's bone marrow pathology. A, The patient's bone marrow smear in May 2016 (Wright stain, 10 × 10). B, The arrow point to primitive monocytes showed in bone marrow smear in May 2016 (Wright stain, 10 × 100). C, The arrow point to primitive monocytes in bone marrow smear was negative with POX in May 2016 (peroxidase stain POX, 10 × 100). D, The patient achieved complete remission after treatment with dasatinib for 2 months (Wright stain, 10 × 100).

**Figure 2:**
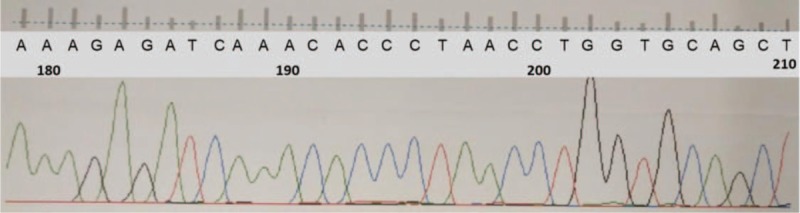
The patient's molecular analysis. The patient's molecular analysis revealed BCR/ABL1 positive without ABL1 mutations in May 2016.

**Figure 3:**
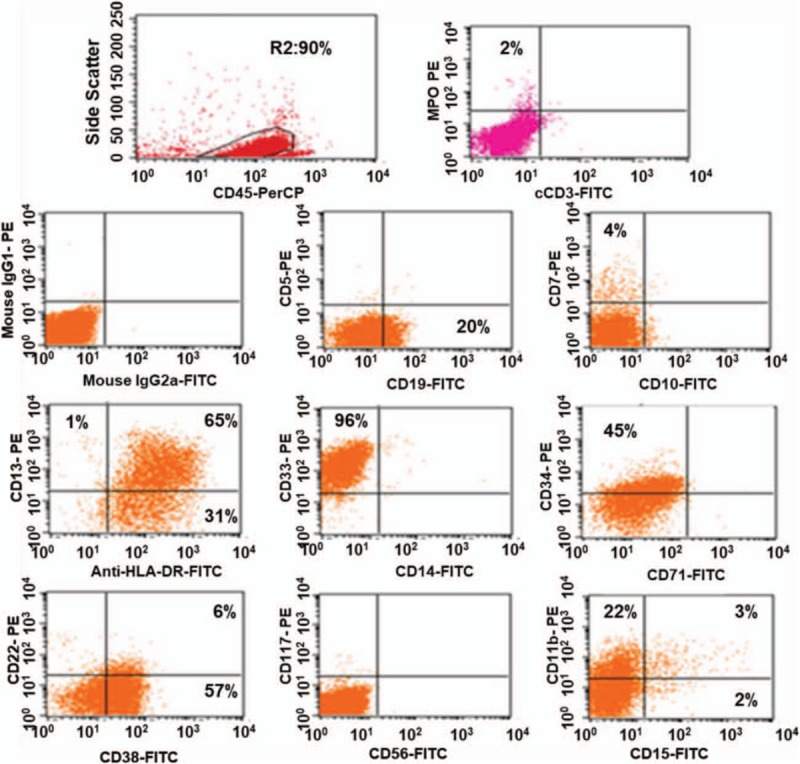
The patient's flow cytometry analysis. Leukemia cells expressed CD13, CD33, CD34, CD11b, CD19, HLA-DR, and CD38, whereas MPO was detected negative.

A 46-year-old man came to our department in May 2016, complaining of fever and cough for 2 days. The laboratory findings were as follows: WBC (119.2 × 10^9^/L), granulocyte (2.3%), lymphocyte (32%), monocyte (65.6%), basophils (0.1%), hemoglobin (98 g/L), platelets (16 × 10^9^/L). His bone marrow smear demonstrated 1 population of blasts (primitive monocytes 63.5%) (Fig. 1), peripheral blood smear found primitive monocytes with absence of basophils. The karyotype analysis of bone marrow cells revealed 46, XY, *t* (9; 22). Molecular genetic analysis showed BCR-ABL1 (p190) positive without ABL1 kinase domain mutations (Fig. 2). No mutations of *FLT3-ITD*, *NPM1*, *C-kit/D816*, *CEBPA*, *PML-RARa*, *MLL*, or *CBFβ/MYH11* were found. Flow cytometry analysis demonstrated 1 population of leukemia cells, expressing CD13 (66%), CD33 (96%), CD34 (45%), HLA-DR (96%), CD38 (63%). CD11b, CD19, CD5, CD7, CD22, CD117, CD10, CD14, CD71, CD56, CD15, cCD3, and MPO were detected negative (Fig. 3) and so was cCD79a in further examination. Physical examination showed no evidence of splenomegaly. According to morphology, immunology, cytogenetic, and molecular criteria, the patient should be diagnosed as Ph+ AML due to no antecedent hematological anomaly. He experienced tumor dissolved syndrome, acute kidney injury, acute heart failure, pulmonary infection, and perianal ulcer combining infection events. After continuous renal replacement therapy and anti-infective therapy, most organ functions returned to normal. We then gave him dasatinib (100 mg/day) considering that he still had a serious perianal ulcer combining infection. The patient had no significant side effects except leukopenia and the need for blood transfusion to support the treatment. Two weeks later, his bone marrow examination demonstrated the status of nonremission. The dosage of dasatinib was then added to 140 mg/day for the next 3 weeks, and a complete remission was achieved. The BCR/ABL1 fusion transcript rate was 12.1%. The dosage of dasatinib was then reduced to 100 mg/day because of leukocyte reduction. After 2 months’ treatment with dasatinib in total, the patient achieved a complete cytogenetic response. Since the patient's serious perianal ulcer improved at that time, so he began to receive 2 cycles of chemotherapy with IA (idarubicin 8 mg/m^2^, day 1–3; cytarabine 100 mg/m^2^, day 1–7) in August, 2016, and finally achieved a complete molecular remission.

## 3 Discussion

In general, Ph+ AML as a high-risk leukemia is assumed although there are no systematic data on outcome. So it was a rare disease and CML primary in myeloid blast crisis. In addition, the distinction between this rarely occurring Ph+ AML and CML primary in blast crisis could be difficult. Furthermore, there were continuing arguments on whether treatment with tyrosine kinase inhibitor (TKI) alone was better than chemotherapy. There were currently no sufficient clinical evidences showing that chemotherapy alone as first-line therapy was superior to TKI.

A very important hallmark of CML was the concomitant basophils accounting for ≥2% of WBC. In this case of Ph+ AML, however, the examination of peripheral blood demonstrated basophils with a rate of 0.1% in WBC. The low level of basophils cells was more conducive to support Ph+ AML. Depending on the current literature, 70% of de novo Ph+ AML patients expressed the Philadelphia chromosome in all metaphases.^[9]^ However, CML-BC is characterized by the presence of the Philadelphia chromosome in 100% of all metaphases. In addition, the most frequent additional chromosome aberrations included trisomy 8, which is an additional Ph chromosome, trisomy 19, and isochromosome 17q in CML-BC.^[10,11]^ These chromosome aberrations were less common in Ph+ AML.^[12]^ As for our patient, we did not detect any of the additional abnormal chromosomes. The BCR/ABL1 fusion protein (p210) was observed in 95% of CML, whereas the p190 was rare in CML. A review showed p190 was found similar to p210 in Ph+ AML, and proposed that the p190 transcript was in favor of the diagnosis of Ph+ AML.^[9]^ It was worth noting that ABL1 mutations were more common in CML-BC, with a frequency as high as 80%.^[13–15]^ The ABL1 mutations have not been reported in Ph+ AML patients.^[3]^ The examinations of our patient showed the existence of p190, whereas the absence of ABL1 kinase domain mutations suggesting that *ABL1* mutations may be an important biomarker for the identification of both Ph+ AML and CML-BC. Bacher et al^[16]^ reported that *BCR/ABL1*, which belongs to the group of class I mutations, conferred the cells a proliferative advantage. The appearance of BCR/ABL1 in AML has been described in combination with different class II aberrations such as CBFβ/MYH11, RUNX1/RUNX1T1, PML/RARa, and NPM1. Neuendorff et al^[17]^ showed a case of BCR/ABL1^+^ MLL/AF6^+^ AML without NPM1 aberrations. MLL rearrangements were mostly considered as class II aberrations.^[18]^ In this patient, we did not find class II aberrations as mentioned above. Previous data suggested that deletion of antigen receptor genes (*IGH*, *TCR*), *IKZF1*, and/or *CDKN2A* may support the diagnosis of de novo disease versus blastic phase of CML.^[19]^ Unfortunately, we did not have a line of the related checks.

BCR/ABL1 fusion protein disturbed downstream signaling pathways, causing enhanced proliferation, differentiation arrest, and resistance to cell death.^[20,21]^ TKIs were the most successful class of molecular targeted therapy for CML, but there were rarely Ph+ AML patients benefit from TKI therapy.^[3,6]^ Neuendorff et al^[17]^ suggested that treatment with a single molecularly targeted (TKI) agent might not be sufficient for disease control of Ph+ AML, so they thought that TKI therapy could not be routinely recommended as a part of first-line therapy. The optimal therapy for Ph+ AML had not been established. However, TKI as a part of salvage therapy is a reasonable approach. As far as our patient was concerned, he experienced multiple organ dysfunction syndrome and severe infection, and he could not tolerate conventional chemotherapy. TKI was suggested to be possible a safe and effective way for Ph+ AML patients who were in poor physical condition and unable to receive chemotherapy immediately. And our patient had no side effects during dasatinib treatment. Furthermore, it was not clear whether to use imatinib, dasatinib, or nilotinib and had the risk of clonal selection in favor of a BCR-ABL-negative subclone. But at that time, he had to control leukemia because of high leukemia burden and avoid aggravating the original infection by attempting to use dasatinib alone. It has been proved that the patient has achieved complete remission. The patient was subsequently treated with haploidentical allogeneic stem cell transplantation after 2 combinations of chemotherapy. And now he is still alive in complete remission. So we believed that TKI could be used as an induction therapy for that could not tolerate chemotherapy. In addition, TKI could be used a salvage chemotherapy after chemotherapy, or as a maintenance therapy or a bridging to transplant after a remission has been achieved.

## 4 Conclusions

In conclusion, we suggested those features: in the absence of basophils, BCR/ABL1+ without *ABL1* mutations may support the diagnosis of Ph+ AML. This case provided evidence for dasatinib treatment to be an effective and tolerable single agent for patients with poor physical condition. It may serve as a replacement therapy, followed by chemotherapy, to help patients achieve complete molecular remission with less adverse events.

## Acknowledgments

The authors would like to thank the patient who took part in this study. A written informed consent for publication has been obtained from the patient.

## Author contributions

**Conceptualization:** Peipei Xu.

**Data curation:** Miaoxin Peng.

**Methodology:** Chaoyang Guan, Pinhao Xie, Cuiying Yuan.

**Writing – original draft:** Dangui Chen, Bing Chen.

**Writing – review and editing:** Dangui Chen, Xiaoyan Shao, Bing Chen.
